# Consumer Motivations for Adopting Omnichannel Retailing: A Safety-Driven Perspective in the Context of COVID-19

**DOI:** 10.3389/fpubh.2021.708199

**Published:** 2022-01-13

**Authors:** Feng Liu, Mingjie Fang, Lanhui Cai, Miao Su, Xueqin Wang

**Affiliations:** ^1^Business School, Shandong University, Weihai, China; ^2^Department of Logistics, Service & Operations Management, Korea University Business School, Seoul, South Korea; ^3^Department of International Logistics, Chung-Ang University, Seoul, South Korea

**Keywords:** COVID-19, omnichannel, protection motivation theory, adoption behavior, health anxiety, retail services

## Abstract

This study aims to understand the influence of COVID-19 on consumers' fears and self-protection motivations. Furthermore, the study seeks to understand the effects of these fears and motivations on consumers' intentions to use omnichannel retailing. A modified theoretical model is proposed by integrating protection motivation theory (PMT) and extending the extended parallel process model (E-EPPM). A total of 398 valid questionnaires are collected and used for further structural equation modeling analysis. The results suggest that the perceived severity, perceived vulnerability, and health anxiety positively impact perceived fears surrounding COVID-19. Furthermore, it is found that perceived fear, self-efficacy, and response efficacy will affect the protection motivation of consumers and ultimately contribute to their behavioral intention to use omnichannel retailing. The findings theoretically enrich the research on COVID-19, PMT, and E-EPPM and empirically provide managerial implications for omnichannel retail service providers.

## Introduction

In recent years, retail service providers have been witnessed to actively integrate and align physical and digital resources, supply chains, and logistics networks in pursuit of a new business model, namely, omnichannel retailing (e.g., Apple, Suning, and Starbucks) (1–3). It is considered an innovative practice to provide consumers with more reliable and seamless channel-agnostic services (4–7). Admittedly, the adoption of multichannel retail services that do business in both online and offline channels has catalyzed better firm performance over the past decades (8). However, the channels are relatively parallel in such settings, which means channel migration in end-to-end shopping activities (from pre-shopping to post-shopping) often encounters barriers. For example, a product bought from the online channel cannot be returned through the offline channel. With the advent of omnichannel retail services, such boundaries can be erased by enabling consumers to choose their preferred end-to-end shopping method. The tenet is that, in any event, no channel barrier would be anticipated by consumers at all stages of the shopping journey (i.e., the interaction between consumer and service provider), which includes, but is not limited to, the process of selection, evaluation, procurement, and after-sale services. In an omnichannel setting, channels are integrated and can be freely chosen by consumers. For instance, one can place an order on the website or by visiting a physical store. After the order is submitted, the buyer can choose home delivery or to collect the product by visiting a service point, and the buyer can also request a pick-up service or visit a service point to complete the return personally if he/she need to return the product (9–11).

Scholars have revealed that omnichannel retailing can benefit stakeholders in various ways. From the service provider's perspective, the operations of omnichannel retailing can translate to increased sales, enhanced revenue, and profitability (4, 12–14). For example, the transformation to omnichannel creates brand equity, such as positive word-of-mouth (15), which potentially attracts more engaged customers. Also, relationships with the existing customer base may deepen due to improved shopping experiences, such as reduced uncertainty and greater agility, leading to better customer retention (16–18). From the customer's perspective, omnichannel retailing can contribute to consumers' opportunity cost savings. For example, omnichannel retailing allows consumers to browse, feel and assess products at their convenience through physical or virtual showrooms (13). Also, consumers' hedonic benefits can be realized through engagement in omnichannel shopping. For example, they may consider arranging purchases and pick-ups according to their preferences a pleasurable activity. Research on omnichannels has been considerably surging because it is especially relevant (8, 19, 20). However, consistent with the review of Mishra et al. (21), it should be noted that few studies have focused on the cognitive dimensions of consumer decision-making in adopting omnichannel retailing. In addition, as they claimed that “*we could not find a single review paper linking consumer decision making with behavioural theories*,” the theoretical foundation related to the consumer perspective is rather underdeveloped.

Moreover, since the massive shock of the ongoing pandemic has changed the business environment and disrupted the retail industry (22–26), we argue that it is necessary to consider the role of COVID-19 in our research model. Indeed, literature on changes in consumer behavior is surging (10, 11, 27–29). Some scholars have reported that COVID-19 has led consumers to panic buy, and they investigated the drivers of this phenomenon (27, 30). Some have argued that consumers' safety and health concerns have catalyzed their dependence on contactless shopping technologies, such as using augmented reality technologies in the information acquisition process (31), live-stream shopping in purchasing (32), and robot or drone deliveries in last-mile logistics (9). Others focused on how the social properties of shopping have been affected by the pandemic. For instance, Wang et al. (28) predicted that COVID-19 would result in the emergence of a massive community of “lonely” consumers who were deeply enveloped in social isolation. Wang et al. (29) analyzed consumer privacy protection in the face of rapidly emerging online shopping. However, to date, knowledge on channel switching behavior under COVID-19 is rather underdeveloped. Specifically, most of the current literature is based on single-channel or multichannel settings, e.g., from brick-and-mortar to online shopping (33), and these arguments may be ill-suited in an omnichannel context.

We aim to fill the knowledge gap by developing a theoretically driven model to identify the antecedents of consumers' adopting omnichannel retailing during the COVID-19 pandemic. In this study, protection motivation theory (PMT) (34) and the extension of the extended parallel process model (E-EPPM) (35) are introduced as theoretical lenses to build the conceptual framework. PMT and E-EPPM are chosen because they consider not only the evaluation of the technology, such as self-efficacy and response efficacy, but also how the external environment creates changes in their intentions to use the technology. The theories are proposed because of their appropriateness for studying the adoption behavior of technologies and COVID-19 research (36–38). This study makes the following academic contributions. First, our research empirically enriches the omnichannel literature by focusing on a consumer behavioral perspective, which is different from past studies mainly located at the organizational level (39, 40). Second, we also contribute to the literature by extending the understanding of the determinants of consumer decision-making in the omnichannel context. By conceptualizing the PMT and E-EPPM model, we explore how consumers' intentions to use omnichannel shopping can be motivated by irrational factors (e.g., fear of COVID-19 and health concerns). This approach is distinct from previous research that has primarily investigated the determinants of consumers' behavioral intentions to use omnichannel retailing through rational-based decisions (5, 41).

The remainder of this study proceeds as follows. The theoretical foundation and hypotheses are presented in the next section. Section Methodology describes the research methodology and data collection. Next, section Results and Discussions covers the data analysis as well as hypotheses testing. The results are then presented and discussed, and section Conclusions summarizes the contributions, limitations, and recommendations for future research.

## Literature Review and Hypotheses Development

### Retailing Channels

Since the last two decades, the retailing industry has experienced a “*single*-*multi*-*omni*” evolution process. “*Single*” refers to retail service providers that sell products or services to customers through only one distribution channel. In this regard, the single-channel model may include the traditional business model (e.g., brick and mortar stores, catalogs, mail-order) or e-commerce (i.e., online stores) (42, 43). A “*multi*” retailer is a firm that adopts a hybrid sales structure by utilizing a variety of different channels as additional service options from their traditional sales structures (4, 44). Therefore, by definition, the difference between the single-channel and multichannel is the number of channels available to consumers. In recent years, both traditional retailers (e.g., Best Buy and Walmart) and online retailers (Amazon and Xiaomi) have been extending their business from “*single*” to “*multi*,” and it has been reported that such strategic changes are helping their financial performance (8). However, it should be noted that channels within the multichannel retailing setting are relatively parallel; that is, the design and operations of these channels are rather independent. For example, transaction data and customer management are not well-integrated, the information transition in different channels is occasionally mismatched, and the “touch-and-feel” is hindered from the online shopping context (45, 46). In this regard, being “*omni*” has obtained much attention as a new business model aimed at integrating all available channels within a firm and eliminating the barriers among these channels (47). Specifically, omnichannel is defined as “*a unified approach that manages channels as intermingled touch points to allow consumers to have a seamless experience within an ecosystem*” (4, 10, 11, 28, 29, 46). In the omnichannel context, consumers are not limited by the channels' characteristics and can migrate across them without any information loss or reiteration.

However, implementing an omnichannel practice is not without difficulties. Some studies have identified a variety of challenges and opportunities for firms' transforming to omnichannels. For example, to operate consistently, “*omni*” requires the fundamental alignment of firms' strategies (18, 48), whereas, in practice, misalignment happens due to conflicts of interest between channels, organizational silos, reduced communication, the isolation of databases, and low logistics service quality (47–51). Despite the significance of these studies, most current studies lack a theory guide, with theoretical research from a consumer behavior perspective only, surprisingly, just starting to emerge. Table 1 summarizes some representative research that is theoretically guided. Some studies have examined channel integration, resource configuration, and operational quality under omnichannel retailing from the view of dynamic capabilities (47, 51), social exchange theory (15), and resource-based theory (53, 54). However, few studies have been conducted to explain consumers' usage behavior, which motivates this study.

**Table 1:** Selected theory-based studies on omnichannel retailing.

Source*	**Theory**	**Method**	**Key findings**
Cao and Li (52)	Innovation diffusion theory	Public data	Channel integration is the key to omnichannel retailing. Firms should develop a higher information technology capability, an open capital market, and a low industry concentration to improve channel integration.
Hossain et al. (47)	Dynamic capabilities theory	Interview and survey questionnaire	Organizational silos are a major obstacle, and firms are under pressure to ensure integration quality. Improved channel-service configuration, content consistency, process consistency, and assurance quality can help overcome this difficulty.
Hüseyinoglu et al. (51)	Dynamic capabilities theory	Survey questionnaire	Operational logistics service quality plays an important role in a successful omnichannel strategy. Firms should reinforce channel integration and ensure consistency.
Juaneda-Ayensa et al. (41)	Unified theory of acceptance and use of technology	Survey questionnaire	Personal innovation, effort expectancy, and performance expectancy are important determinants of consumer purchase intention in the omnichannel setting.
Lee et al. (15)	Social exchange theory	Survey questionnaire	Engaging customers is challenging, and the breadth of channel-service choice, the transparency of channel-service configuration, and content and process consistency are critical to solving this problem.
Shen et al. (46)	Wixom & Todd model	Survey questionnaire	Channel service transparency, content consistency, and process transparency determine the success of firms' omnichannel strategies.
Luo et al. (53)	Resource-based theory	Public data	There is a positive relationship between firms' information technology applications and cross-channel capabilities, and such association is positively moderated by financial resources.
Song et al. (54)	Resource-based theory	Survey questionnaire	Higher levels of supply chain integration result in a better performance of omnichannel retailers, and supply chain integration capabilities can be improved from information, process, and organization integration capability.
Xu and Jackson (5)	Theory of planned behavior; commitment–trust theory	Survey questionnaire	Consumers' adoption behaviors in omnichannel retailing are determined by perceived behavioral control, perceived risk, and price advantage.

* *Studies are ordered alphabetically based on the first author's name*.

### Theoretical Background

The theoretical background for this study stems from the protection motivation theory (PMT) (34) and the extension of the extended parallel process model (E-EPPM) (35). PMT theorizes that people's decisions to undertake self-protective actions when facing a noxious event are broadly initiated and maintained through two cognitive appraisal processes: threat appraisal and coping appraisal (34, 55, 56). In other words, whether to adopt the recommended measure to protect themself is contingent on their assessment of the threat magnitude of the event (threat appraisal) and the effectiveness of the recommended measure (coping appraisal). If an event is considered harmful and the recommended coping measures have high perceived efficacy, as well as low costs, they would exhibit a high motivation to use the recommended coping measures to protect themselves from being influenced or, more seriously, being injured (57).

Threat appraisal refers to one's assessment of the perceived severity and vulnerability, which examines the potential severity of an event (i.e., perceived severity) and what (mostly negative) consequences it would probably lead to (i.e., perceived vulnerability). A coping appraisal consists of three constructs, namely, self-efficacy, response efficacy, and response costs, which refers to one's evaluation of the following: (a) beliefs or confidence in their ability to engage in the coping response (i.e., self-efficacy); (b) the degree of perceived efficiency of the recommended response in avoiding the threat (i.e., response efficacy); and (c) costs would incur when the recommended response is taken (i.e., response costs). To illustrate: when considering whether to contribute to reducing greenhouse emissions through the use of public transportation, an individual would evaluate the risk caused by greenhouse emissions (*perceived severity*) and the individual's susceptibility to the risk of greenhouse emissions (*perceived vulnerability*). At the same time, the individual would also assess their capability to use public transport (*self-efficacy*), the effectiveness of public transport in reducing air pollution (*response efficacy*), and the costs associated with using public transport (*response costs*).

There is consistent evidence that PMT is a robust model for explaining and promoting health and safety-related attitudes and behaviors (36, 38), and “*can be applied to any threat that an individual can assess and respond to accordingly”* (55). In fact, it has been widely utilized in a diverse disciplines, such as medical care (36, 58, 59); environmental sciences (60–62); information security (63–65); and transportation (66–68). Recently, it has been observed that extensive epidemiological research employing PMT to study behavioral or psychological changes related to COVID-19 has been conducted. For example, the fear of traveling (69) and social distancing (70, 71). Therefore, we believe that PMT could be used as the foundation of our research model. Consistent with much PMT-related literature that excluded response costs from the research framework [e.g., see (33, 72)], responses costs were excluded in our conceptual framework. This approach was taken since numerous studies have suggested that omnichannel retailing is a novel technology that forms a seamless retail world for consumers, and the adoption of such shopping methods does not require additional efforts and does not cause advantage losses to other shopping methods (11, 29, 73).

Indeed, the evolving COVID-19 pandemic has caused much psychological fallout, the most prominent consequence of which can be concluded as fear. Although PMT includes, to some extent, the effect of affective assessments on protection motivation, the role of fear as an antecedent is ignored. This drawback can be addressed by introducing EPPM (74), and the cognitive appraisal theory of emotions (75). It holds that an individual's threat appraisal of an event is triggered by fear, and a coping appraisal would be provoked when the degree of fear somewhat exceeds a specific threshold. However, Witte (76) sought to adequately explain the association between perceptions and behaviors by pointing out that the tenets of EPPM (i.e., “putting the fear back into fear appeals” and “heavy focus on cognition”) can lead to biased outcomes and the failure. Therefore, the extension of the extended parallel process model (E-EPPM) is chosen as part of our conceptual framework to provide a better theoretically-grounded explanation. Different from EPPM, E-EPPM distinguishes itself by redefining constructs such as perceived risk. Also, a new component, i.e., anxiety, is considered a critical factor in the model. Since fear and anxiety are two different emotions and have distinct central thematic associations, we believe that the role of anxiety as an antecedent also requires examination (77). Moreover, since extensive research has suggested a tight linkage among COVID-19, fear, anxiety, and behaviors (37, 78, 79), we argue that E-EPPM is more suitable for this context defined by the COVID-19 pandemic. The study's dependent variable is consumers' behavioral intentions, and, in the context of this research, it is defined as the aim and plan of repeatedly using omnichannel retail services for their shopping activities (10, 11).

**Figure 1:**
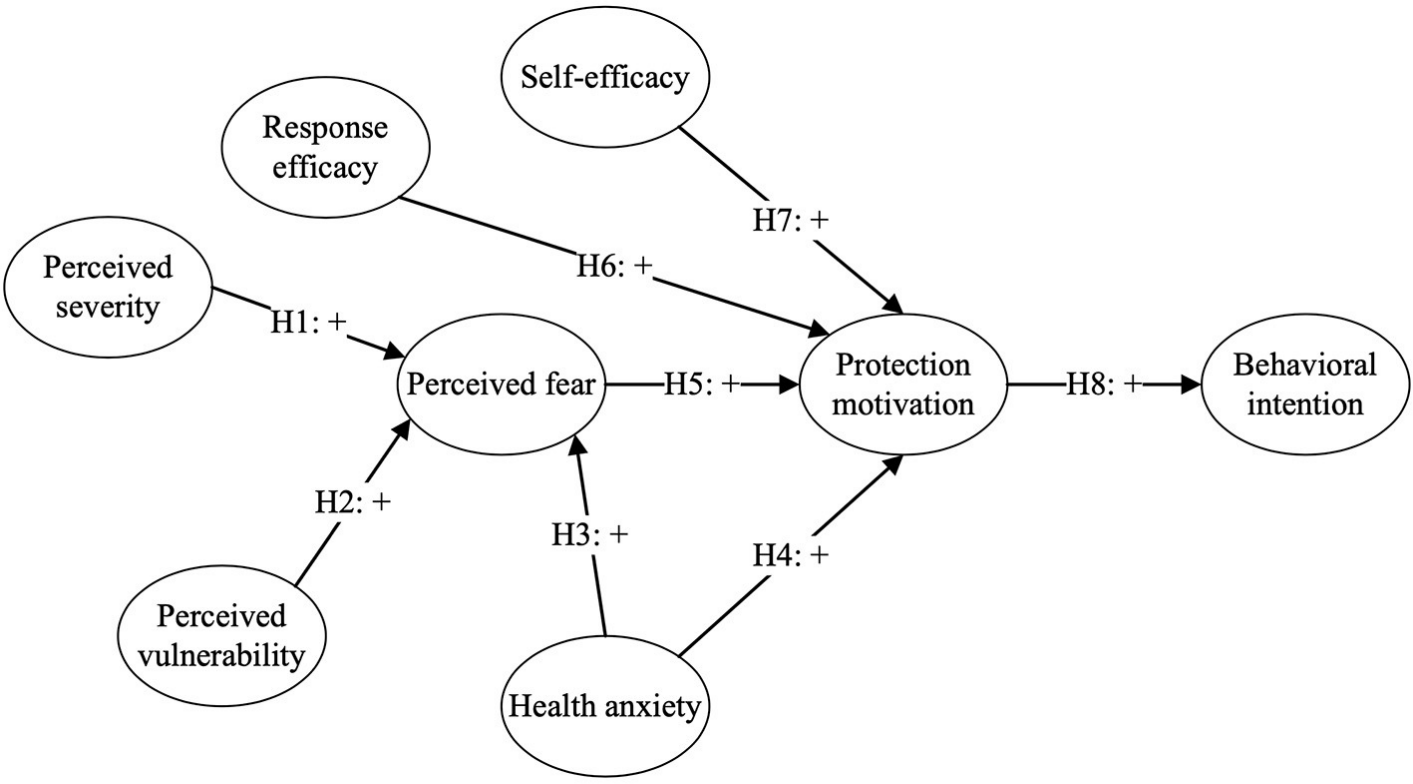
The theoretical framework.

Drawing on the interpretation of the theories, we propose a modified theoretical model to investigate the antecedents influencing consumers' behavioral intentions to use omnichannel retailing by incorporating the theoretical lens of PMT and E-EPPM, which is depicted in Figure 1.

### Hypotheses Development

In this study, perceived severity is defined as an individual's evaluation of the negative consequences of the COVID-19 pandemic, while perceived fear refers to their psychological distress due to these consequences (80, 81). Earlier studies have confirmed a positive relationship between perceived severity and perceived fear (82–84). In the context of COVID-19, its impact on people's health and livelihoods is considered a possible consequence that leads to their perceived fear. Indeed, as of December 28, 2021, more than 280 million cases, including over 5 million COVID-19 deaths, have been reported worldwide (85). Critically, the ongoing pandemic is still yet to be effectively controlled, with significant outbreaks occurring from time to time in certain areas (e.g., South Korea, India, and Colombia). Thus, we argue that such a rapidly evolving situation will likely evoke people's fear-based emotions. Therefore, the following hypothesis is proposed:

H1. The perceived severity of COVID-19 positively influences the perceived fear of COVID-19.

Perceived vulnerability refers to an individual's evaluation of the probability that the depicted event may potentially harm them. According to an empirical study conducted in Belgium, significant results reflected that respondents perceived themselves as susceptible to COVID-19 (86), noting that, while it is generally accepted that the less time people stay outdoors, the safer they will be (i.e., stay-at-home policies), the findings were not influenced by respondents' commuting distances. This outcome is supported by a cross-country study, which indicated that respondents perceived threats, as well as fear of being affected by COVID-19, as high (87). Thus, the following hypothesis is proposed:

H2. The perceived vulnerability to COVID-19 positively influences the perceived fear of COVID-19.

Despite perceived severity and vulnerability being included in our model for the COVID-19 threat appraisal, the study also considers the role of anxiety because E-EPPM posits that anxiety is critical to an individual's threat perception and plays an essential role in stimulating protective responses (35). Since COVID-19 is considered a health crisis, we narrow the variable anxiety to a more health-focused perspective, i.e., health anxiety. In this study, health anxiety is defined as a person's apprehension or concern about their health status in the context of COVID-19 (79, 88). Many studies have indicated that the person who is always suspicious that that have, or are suffering from, a serious illness tends to have a higher perceived fear (89–91). Moreover, according to So et al. (77), anxiety is another critical factor, other than perceived fear, that is expected to have an essential impact on protection motivation. Along the same lines, Knowles and Olatunji (92) supported this view by illustrating a causal relation between anxiety and safety behaviors. On this basis, the following is hypothesized:

H3. Health anxiety positively influences perceived COVID-19 fear.
H4. Health anxiety positively influences protection motivation.

Many researchers believe there is a positive correlation between perceived fear and protective motivation (37, 93–95). For example, Yuen et al. (96) reported that panic buying occurred globally at the outbreak of COVID-19 because the sudden emergence of the disease made people feel threatened and induced fear of the unknown, thus somehow motivating them to stockpile large amounts of supplies as their protective behavior. In addition, the consumption behavior relating to the siginificant surge in the consumption of personal protective equipment out of fear of contracting COVID-19 was witnessed, and the increasing trend was strongly tied to the pandemic (93). Therefore, the following hypothesis is proposed:

H5: Perceived COVID-19 fear affects protection motivation positively.

To reiterate: two components are considered in the coping appraisal, i.e., self-efficacy and response efficacy. In the context of this study, self-efficacy is defined as the expectancy of an individual's ability in performing omnichannel retailing as the recommended coping behavior, while response efficacy is the degree to which an individual believes that using omnichannel retailing will be an effective means to avoid being harmed or infected by COVID-19. Consumers who perceive the use of omnichannel retailing as useful in protecting themselves from COVID-19 are likely to be more motivated to take protective action. Furthermore, consumers who assess themselves as being able to utilize omnichannel retailing are also more likely to take protective measures. PMT predicts that both self-efficacy and response efficacy will positively impact protection motivation (94). Therefore, the following hypotheses are proposed:

H6. Self-efficacy positively influences protection motivation.
H7. Response efficacy positively influences protection motivation.

Protecting motivation lies at the core of PMT (34, 55). However, since the protection motivation should precede actual action of using the recommended coping behavior in the pandemic, there is a tendency to explore whether such motivation would transfer to consumers' actual behavior in using omnichannel retailing. According to the World Health Organization, it is important to take precautions, such as staying at home, social distancing, and refraining from spending long periods in crowded environments (97). We argue that consumers' protection motivation is positively related to their use of omnichannel retailing since it can support these suggestions in several ways. For example, by utilizing the flexible options of purchase and pick-up, consumers may use augmented reality technology to select and experience items on their mobile devices. They may then choose online delivery or collect them during low customer flow hours (buy online–in-store-pick-up), thus avoiding extra time and effort, thereby reducing close contact with others. Moreover, in the context of omnichannel retailing, consumers are also allowed to use multiple channels. For example, consumers can ask the retailer to collect the return or randomly bring the items to any retail store at their convenience. This scenario further reinforces the role of omnichannel retail in helping consumers choose the proper engagement method to avoid exposure to relatively dangerous environments. In addition, many studies have reported that COVID-19 has resulted in panic buying because people were motivated to hoard necessities as their coping behavior (27, 30). In this regard, consumers may be more likely to use omnichannel retail services because they have better dynamic information processing capabilities for external conditions (7), and by extension, higher supply chain resilience. Such an improved business model would encounter fewer shortages and, thus, better meet consumer demand. Based on the above discussion, the following hypothesis is proposed:

H8. An increase in consumers' protection motivation increases their behavioral intention to use omnichannel retailing.

## Methodology

### Survey Design and Measurement Items

A survey methodology that is consistent with existing quantitative protection motivation and omnichannel retailing research was adopted to test the hypotheses empirically [see Bulgurcu et al. (98), Ifinedo (99), Lee et al. (15), and Xu and Jackson (5)]. The questionnaire consists of three sections. Section Introduction was the description, including the background and objective of the survey and an explanation of omnichannel retailing. We sought to ensure common understanding by focusing more on providing straightforward examples describing the usage scenario rather than technical terms to explain the working mechanism of omnichannel retailing. Section Literature Review and Hypotheses Development contained a survey of social-demographic characteristics, including gender, age, education, monthly income, and the most frequently used payment method in daily life. Section Methodology contained items for measuring the eight latent variables in our conceptual model: perceived severity, perceived vulnerability, perceived fear, health anxiety, self-efficacy, response efficacy, protection motivation, and behavioral intention. A screening question was added at the beginning of section Methodology to ensure that respondents fully understood the instructions and answered the questions carefully. It was a multiple-choice question that asked respondents to select the example that was not omnichannel retailing. Furthermore, another question requiring respondents to select “*strongly agree*” was also inserted in section Methodology.

All items were developed from relevant previous research, and appropriate modifications were made in consultation with a review panel (e.g., three practitioners from the omnichannel retailing industry, two professors whose research interests are retailing, service operations management, and consumer behavioral research, and one doctors) to ensure content validity. A total of 28 measurement items were adapted to operationalize the eight constructs (see Table 2). Specifically, three items were adopted to measure perceived severity (100, 101); a four-item scale was chosen from Bashirian et al. (80) to detect perceived vulnerability; four items measured perceived fear; while respondents' health anxiety was selected for measurement by three items (102, 103). Self-efficacy and response efficacy were measured using three and four items, respectively (69, 104–106). Three measures for behavioral intention were adopted from Chang and Chen (107). A seven-point Likert scale ranging from 1 = “*strongly disagree*” to 7 = “*strongly agree*” was used to evaluate these items.

**Table 2:** Scale development.

**Construct**	**Measuring items**	**Source**
Perceived severity (PS)	*Strongly disagree (1)/Strongly agree (7)*	(100, 101)
	PS1. I find COVID-19 is a serious disease.	
	PS2. I think the COVID-19 outbreak will continue for at least the following 6 months.	
	PS3. It would be serious if I get sick from COVID-19.	
Perceived vulnerability (PV)	*Strongly disagree (1)/Strongly agree (7)*	(80)
	PV1. I think I am at risk of getting COVID-19 in given circumstances.	
	PV2. It is possible for me to be infected by COVID-19.	
	PV3. Most people I know are vulnerable to COVID-19.	
	PV4. It is likely that my family and friends would be infected by COVID-19.	
Perceived fear (PF)	*Strongly disagree (1)/Strongly agree (7)*	(102)
	PF1. It makes me uncomfortable to think about COVID-19.	
	PF2. My hands become clammy when I think about COVID-19.	
	PF3. I cannot sleep because I am worrying about getting COVID-19.	
	PF4. My heart races or palpitates when I think about getting COVID-19.	
Health anxiety (HA)	*Strongly disagree (1)/Strongly agree (7)*	(103)
	HA1. I am less likely to notice sensations/changes in my body than other people.	
	HA2. It's hard for me to free myself from concern about my health.	
	HA3. I sometimes suspect that I have a serious illness.	
Self-efficacy (SE)	Strongly disagree (1)/Strongly agree (7)	(69, 104)
	SE1. I believe that making an effort to reduce the spread of COVID-19 is worthwhile.	
	SE2. I believe that I can make contributions to the fight against COVID-19.	
	SE3. I have the skills required to prevent being infected by COVID-19.	
Response efficacy (RE)	*Strongly disagree (1)/Strongly agree (7)*	(105, 106)
	RE1. Omnichannel shopping is conducive to avoiding being exposed to COVID-19.	
	RE2. By using omnichannel shopping, the chance of being infected with COVID-19 would be decreased.	
	RE3. Omnichannel shopping works to prevent the spread of COVID-19.	
	RE4. Omnichannel shopping is an effective measure for preventing COVID-19.	
Protection motivation (PM)	*Strongly disagree (1)/Strongly agree (7)*	(94)
	PM1. I think I need omnichannel shopping to protect myself.	
	PM2. I believe that it is necessary to use omnichannel shopping to reduce the probability of exposure to COVID-19.	
	PM3. I believe that I must use omnichannel shopping to reduce the probability of COVID-19 infection.	
	PM4. I believe that others must use omnichannel shopping to reduce the probability of COVID-19 infection.	
Behavioral intention (BI)	*Strongly disagree (1)/Strongly agree (7)*	(107)
	BI1. I would consider omnichannel shopping for my next purchase.	
	BI2. I would list omnichannel shopping as one of my top options.	
	BI3. I would share my positive attitude about omnichannel shopping with people.	

A pretest was administered to 13 native English-speaking participants at the corresponding author's university in South Korea to check the appropriateness of the survey. It was reported that some of the questionnaire items should be improved for more simplicity. Additionally, it was suggested that section Introduction should contain more information to help respondents better comprehend omnichannel retailing, such as more examples regarding the usage scenario differences from other channels. Therefore, some revisions were made to the phrasing of the measures and the description sheet.

### Data Collection

The questionnaire was administered to residents in Seoul, Korea. Seoul was selected because the lock-down policy was not implemented during the COVID-19 pandemic. Therefore, outdoor activities were not restricted. Previous evidence highlighted that compatibility and connectivity play a significant role in consumers' perceptions of omnichannel shopping (2, 108). Since lock-down may disrupt some components of the value chain (e.g., the operations of physical stores), it may influence the consumer's understanding of omnichannel retailing, which affects the data quality.

A translation and back-translation approach was used to ensure semantic equivalence and enable the questionnaire to be fully and easily understood by respondents. First, a professional translator was assigned to translate the original English version into Korean. Afterward, the Korean version of the questionnaire was back-translated by another translator who did not see the original version of the questionnaire. After completing the translation process, the authors and translators engaged in discussions to compare both the original and back-translation versions of the questionnaire to identify discrepancies and revise the Korean version of the questionnaire. Finally, the refined questionnaire was generated online on Google Forms, and a QR code generator was used to create a QR code that provides access to the questionnaire.

The survey was conducted simultaneously at five randomly selected shopping areas with high customer flow. They included Myeongdong, Dongdaemun, Hongdae, Apgujeong, and Yongsan. Customers who were willing to participate in the survey were invited to scan the QR code via their mobile devices. Note that respondents' email addresses were automatically collected to email them the coffee gift card. The survey was conducted for 2 weeks in February 2021, from 9:00 a.m. to 9:00 p.m. A total of 579 questionnaires were received. It was discovered that respondents generally took more than 5 min to answer all the questions during the pretest. As a result, respondents who were recorded as finishing the survey too quickly (taking <2 min) were regarded as invalid responses. In addition, responses with incorrect answers to the screening questions were also discarded. Ultimately, 398 questionnaires were effectively used for further analysis, representing a conversion rate of 69%.

## Results and Discussions

### Demographic Statistics

The respondents' demographic profiles, as well as the characteristics of their most frequently used payment methods are shown in Table 3. The proportion of males (52.01%) is marginally larger than females (49.99%). The majority of respondents are either young (60.80% were under 30) or middle-aged (37.19% are between 30 and 49). They also have a high level of education, with most respondents (71.61%) having received a bachelor's degree or above. Next, around 62.06% of respondents have a monthly income between 2.5 and 4.5 million KRW (~US$ 2,250–4,000). Regarding the payment method, the most frequently used by respondents in their daily lives are credit cards (80.65%), followed by mobile devices (11.56%), cash (7.29%), and others (0.5%).

**Table 3:** Respondent demographics and their most frequently used payment method.

**Items**	**Category**	**Frequency**	**Percentage (%)**
Gender	Male	207	52.01
	Female	191	47.99
Age (years)	<20	41	10.30
	20–29	201	50.50
	30–39	116	29.15
	40–49	32	8.04
	>50	8	2.01
Education	High school or below	45	11.31
	Diploma	68	17.09
	Bachelor	239	60.05
	Postgraduate or above	46	11.56
Monthly income (million KRW) (1 million KRW = 899.81 USD*)	No income	32	8.04
	<1.50	46	11.56
	1.50–2.49	42	10.55
	2.50–3.49	126	31.66
	3.50–4.50	121	30.40
	>4.50	31	7.79
Payment method	Cash	29	7.29
	Credit card	321	80.65
	Mobile device	46	11.56
	Others	2	0.5

* *South Korean Won to US dollar conversion - last updated Apr 26, 2021, 13:10 UTC*.

### Measurement Model Assessment

Confirmatory factor analysis is performed to evaluate the reliability and validity of the measurement model fit, and the results are presented in Tables 4, 5. According to Hu and Bentler (109), a good model fit is considered as fulfilling the following criteria: (1) the ratio of the Chi-square value to the degree of freedom (χ^2^/*df* ∈(1, 3)); (2) the Tucker-Lewis index (TLI) > 0.9; (3) the comparative fit index (CFI) > 0.9; (4) the root mean square error of approximation (RMSEA) < 0.08; and (5) the standardized root mean square residual (SRMR) < 0.08. The results of the study show that all the indices are within the cut-off range (χ^2^/df = 2.10, df = 320, CFI = 0.96, TLI = 0.95, RMSEA = 0.05, SRMR = 0.06). Therefore, the model's goodness of fit was supported.

**Table 4:** Confirmatory factor analysis results.

**Construct**	**Item**	**λ**	**AVE**	**CR**
Perceived severity (PS)	PS1	0.85	0.67	0.86
	PS2	0.78		
	PS3	0.83		
Perceived vulnerability (PV)	PV1	0.74	0.69	0.90
	PV2	0.87		
	PV3	0.85		
	PV4	0.86		
Perceived fear (PF)	PF1	0.87	0.74	0.92
	PF2	0.90		
	PF3	0.78		
	PF4	0.88		
Health anxiety (HA)	HA1	0.82	0.62	0.83
	HA2	0.84		
	HA3	0.70		
Self-efficacy (SE)	SE1	0.89	0.69	0.87
	SE2	0.88		
	SE3	0.71		
Response efficacy (RE)	RE1	0.68	0.56	0.84
	RE2	0.76		
	RE3	0.84		
	RE4	0.71		
Protection motivation (PM)	PM1	0.87	0.70	0.90
	PM2	0.80		
	PM3	0.93		
	PM4	0.73		
Behavioral intention (BI)	BI1	0.88	0.64	0.84
	BI2	0.77		
	BI3	0.75		

*Model fit indices: χ^2^/df = 2.10, (p < 0.05, df = 320); CFI = 0.96; TLI = 0.95; RMSEA = 0.05; SRMR = 0.06*.

**Table 5:** Square roots of AVE, MSV, and ASV and correlations of the constructs.

	**AVE**	**MSV**	**ASV**	**BI**	**PM**	**PF**	**RE**	**SE**	**PS**	**PV**	**HA**
BI	0.64	0.56	0.16	**0.80^a^**							
PM	0.70	0.41	0.20	0.75^b^	**0.84**						
PF	0.74	0.18	0.14	0.34	0.43	**0.86**					
RE	0.56	0.41	0.16	0.53	0.64	0.32	**0.75**				
SE	0.69	0.24	0.10	0.38	0.44	0.28	0.49	**0.83**			
PS	0.67	0.18	0.06	0.07	0.20	0.42	0.14	0.13	**0.82**		
PV	0.69	0.20	0.07	0.09	0.16	0.37	0.16	0.11	0.32	**0.83**	
HA	0.62	0.20	0.06	0.04	0.05	0.41	0.09	−0.01	0.25	0.45	**0.79**

a *Square root of AVE values are along the main diagonal*;

b *Correlations of constructs are below the main diagonal*.

In addition to the model fit, the measurement model is also evaluated for reliability, convergent validity, and discriminant validity. Regarding reliability, Hair et al. (110) proposed that the composite reliability (CR) of each construct should exceed 0.70, and standardized factor loadings (λ) should be higher than 0.50, ideally, 0.70. According to Table 3, the CRs of the constructs are all above 0.80. Therefore, the constructs of the measurement model are considered reliable. In addition, as displayed in Table 4, standardized factor loadings varied between 0.68 and 0.93, demonstrating that all measurement items are acceptable. For the convergent validity, scholars have suggested that the constructs' average variance extracted (AVE) should exceed 0.50 (111). Therefore, the convergent validity of the model is confirmed as the average variances extracted (AVEs) in Table 4, which are all above the threshold value.

Regarding discriminant validity, three conditions should be satisfied: (1) the inter-correlation values between constructs should be less than the square root of the AVE, (2) the construct's AVE value should be greater than its corresponding maximum shared variance (MSV), and (3) the average shared variance (ASV) of the construct should be smaller than its AVE (112, 113). As shown in Table 5, all the constructs have fulfilled the above conditions, suggesting confirmation of discriminant validity.

### Structural Model Assessment

**Figure 2:**
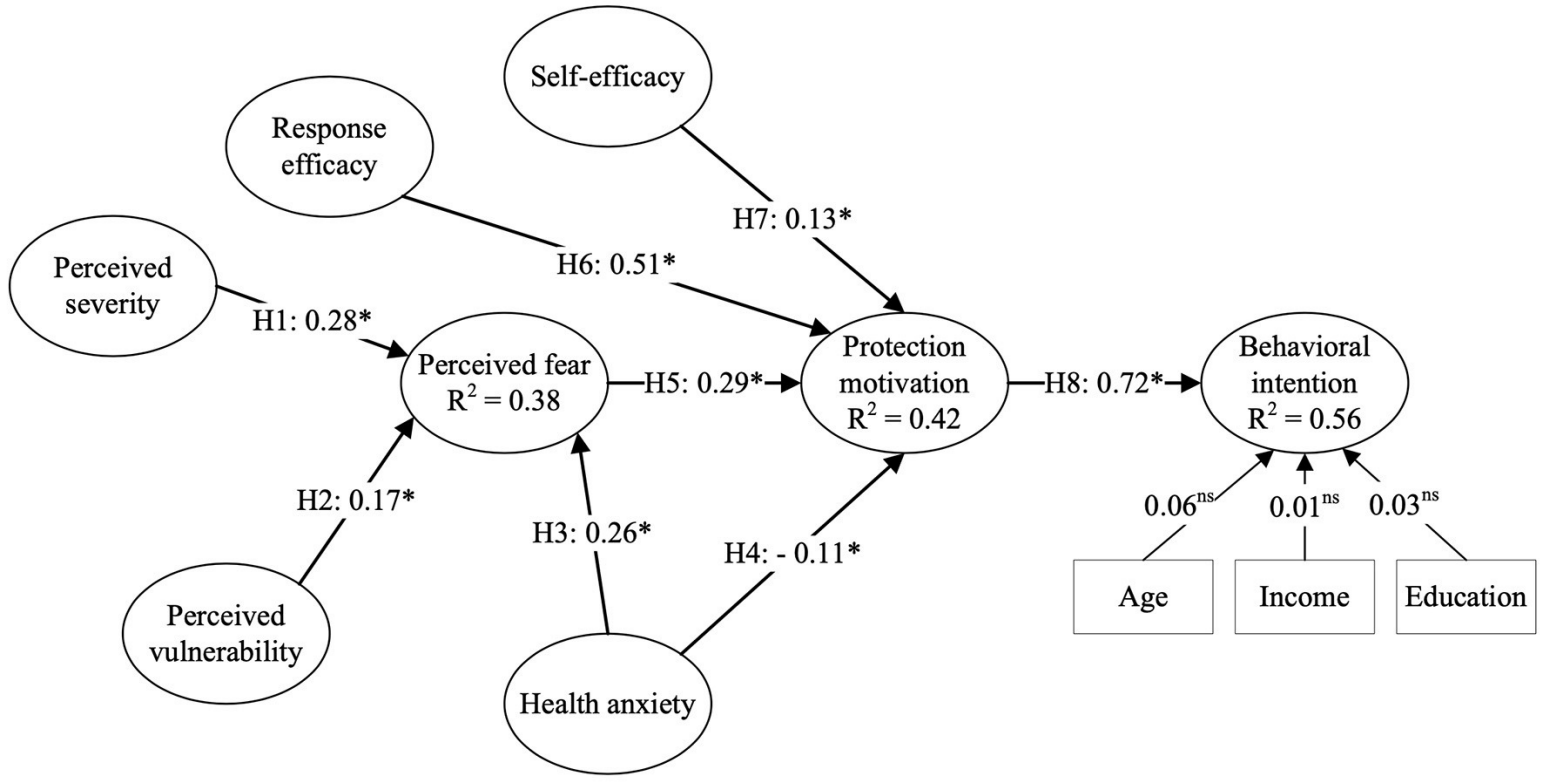
Results of structural model analysis. Model fit indices: χ^2^/df = 1.86, CFI = 0.95, TLI = 0.95, RMSEA = 0.05, SRMR = 0.07. *Indicates *p* < 0.05.

The structural equation model (SEM) is applied to evaluate the structural model. Note that control variables, including age, education, and income, are also added to the model to analyze their effects on consumers' behavioral intentions to use omnichannel retailing. Hypotheses are examined through the significance and the standardized estimated correlations of the constructs. In addition, squared multiple correlations (*R*^2^) are calculated to determine the percentage variance explained by the latent variables. The results are graphically depicted in Figure 2.

Figure 2 shows that the structural model fit was sufficient (χ^2^*/df* = 2.58, CFI = 0.95; TLI = 0.95; RMSEA = 0.06; SRMR = 0.07). Overall, all hypotheses were accepted, except H4, and the relationship between the control variables and consumers' behavioral intentions to use omnichannel shopping was not statistically significant.

Significant positive relationships are found between perceived severity and perceived fear (β = 0.28, *p* < 0.05); thus, H1 was accepted. This outcome is consistent with previous findings in COVID-19 research, which have reported that consumers who feel unsafe due to the pandemic can lead to a greater perceived fear of COVID-19 (114). Also, in line with previous studies (81), H2 is supported because the positive path from perceived vulnerability to perceived fear was significant (β = 0.17, *p* < 0.05). Therefore, consumers assess COVID-19 as a health crisis that makes them susceptible to infection, and this concern contributes to their perceived fear of COVID-19.

Moreover, while the predictor health anxiety significantly affects perceived fear and protection motivation (*p* < 0.05), the path coefficients for the two are 0.26 and −0.11, respectively, thus generating support for H3 but rejection for H4. The acceptance of H3 is expected since the E-EPPM posits that, because people who are health anxious are more sensitive and more afraid of uncertainties than those without health anxiety, they experience higher levels of perceived fear. Interestingly, as displayed in Figure 2, the path loading of health anxiety (β = 0.26) is lower than perceived severity (β = 0.28), implying that there is a consensus on the severity of the COVID-19 pandemic. These antecedents explain 38% of the variance in perceived fear (*R*^2^ = 0.38) collectively. However, the rejection of H4 is unexpected, which is inconsistent with previous findings (77, 92).

According to the rejection of H4 and the acceptance of H5 [perceived fear (β = 0.29, *p* < 0.05)], the indirect effect of health anxiety on consumers' protective motivation to use omnichannel retail services as a response to COVID-19 is found, highlighting the role of perceived fear in explaining consumers' protection motivation.

Furthermore, response efficacy (β = 0.51, *p* < 0.05) and self-efficacy (β = 0.28, *p* < 0.05) had significant positive effects on protection motivation, supporting H6 and H7. The positive relationship between response efficacy and protection motivation is aligned with PMT, suggesting that consumers will be more motivated to adopt omnichannel retail services if they assess themselves as capable of using omnichannel retailing. In addition, the positive association between response efficacy and protection motivation is also coherent with the PMT, which states that consumers who perceive the use of omnichannel retailing as being useful to protecting themselves from COVID-19 are likely to be more motivated to take this as protective actions. Together with perceived fear, these three constructs explain 42% of the variance in protection motivation (*R*^2^ = 0.42). Finally, the behavioral intention was found to be positively and significantly predicted by protection motivation, with a variance explanation of about 56% (*R*^2^ = 0.56). Thus, H8 was accepted.

Notably, no statistically significant linkage was found between the control variables and behavioral intention. This outcome contradicts previous studies. For example, it has been reported that the elderly tend to be more reluctant to new technology than younger people (115). Notwithstanding, this finding confirms that the theoretical constructs are stronger indicators of consumers' behavioral intentions to adopt omnichannel retail services than demographic variables.

### Mediation Test

A bootstrap mediation analysis using a sample size of 5,000 for the bias-corrected confidence intervals was conducted (116) to examine the mediated relationships in the conceptual framework, and the results were presented in Table 6. Overall, the study's model does not contain a zero value in terms of the 95% confidence intervals, confirming the statistical significance of the mediating effects. Perceived fear is found to fully mediate the relationship between consumers' protection motivation and perceived severity (b_ind_ = 0.08, Boot SE = 0.02, *p* < 0.001) and perceived vulnerability (b_ind_ = 0.05, Boot SE = 0.02, *p* < 0.01). Because both the direct effect (*p* < 0.05) and indirect effect (*p* < 0.001) of health anxiety on protection motivation are statistically significant, perceived fear is a partial mediator of the relationship between health anxiety and protection motivation. Moreover, protection motivation is also confirmed as an effective mediator between consumers' behavioral intention to use omnichannel retailing and the constructs of the PMT and E-EPPM. Specifically, response efficacy has the largest indirect effect on behavioral intention (b_ind_ = 0.37, Boot SE = 0.05, *p* < 0.001), followed by perceived fear (b_ind_ = 0.21, Boot SE = 0.04, *p* < 0.001), self-efficacy (b_ind_ = 0.10, Boot SE = 0.06, *p* < 0.001), perceived severity (b_ind_ = 0.06, Boot SE = 0.02, *p* < 0.001), perceived vulnerability (b_ind_ = 0.04, Boot SE = 0.02, *p* < 0.01), and health anxiety (b_ind_ = −0.03, Boot SE = 0.03, *p* < 0.01), respectively. In this regard, the effects of perceived severity, perceived vulnerability, and health anxiety are channeled via dual mediators (i.e., perceived fear and protection motivation).

**Table 6:** Bootstrapping test results.

	**Indirect effect**	**Boot SE^a^**	**Sig**.	**BLLCI^b^**	**BULCI^c^**
PS to PM	0.08	0.02	***	0.04	0.13
PV to PM	0.05	0.02	**	0.01	0.10
HA to PM	0.08	0.02	***	0.04	0.13
PF to BI	0.21	0.04	***	0.14	0.29
PS to BI	0.06	0.02	***	0.03	0.10
PV to BI	0.04	0.02	**	0.01	0.07
HA to BI	−0.03	0.03	**	0.01	0.09
RE to BI	0.37	0.05	***	0.28	0.47
SE to BI	0.10	0.06	***	0.08	0.22

a *Boot SE, Bootstrap standard error*;

b *BLLCI, Bootstrap lower limit confidence interval*;

c *BULCI, Bootstrap lower limit confidence interval*;

* *;p < 0.05*,

** *p < 0.01*,

*** *p < 0.001*.

## Conclusions

### Theoretical Contributions

Theoretically, this study contributes to the existing literature in several ways. First, it enriches the literature on COVID-19 by empirically revealing the pandemic's psychological impact on consumers. The results indicate that the severity and vulnerability of COVID-19 and consumers' health anxiety are significantly associated with perceived fear while also demonstrating that, together with response efficacy and self-efficacy, these factors would trigger their protective motivations. Although the positive impact of health anxiety on protection motivation was not statistically validated (H4), the results highlighted the mediating role of perceived fear in explaining consumers' protection motivations.

Second, this study adds knowledge to the body of PMT and E-EPPM research by proposing and validating a research conceptualization that integrates PMT and E-EPPM to identify the antecedents that influence consumers' intentions to use omnichannel retailing. Specifically, the former considers the influence of coping behaviors, while the latter describes the effect of emotional appeals on omnichannel retailing adoption intentions. The results demonstrate that both PMT and E-EPPM are theoretical perspectives appropriate for this context and provide better insight into consumers' behaviors and decision-making processes concerning the selection of retailing services. Overall, the integrated model explains 56% of the variance in consumers' intentions to adopt omnichannel retail services, which is considered sizeable in the context of consumer behavior research.

Third, this study advances the current understanding regarding the determinants of consumers' intentions to use omnichannel retail services. Despite the critical role of consumer behavior in omnichannel retailing, the theoretical foundation related to the consumer perspective has been discovered to be rather underdeveloped (21). This study identifies that consumers' intentions to use omnichannel retailing can be trigged by their safety concerns; that is, perceived fears evoked by perceived severity, vulnerability, and health anxiety would drive them to use omnichannel retailing services as coping measures. Furthermore, factors such as self-efficacy and response efficacy are found to be attributed to consumers' intentions to use omnichannel retailing. By doing so, this study echoes Mishra et al.'s [(21), p.161] call for more research on the predominance of perceptual dimensions of consumers' decision-making in omnichannel retailing.

### Managerial Implications

The managerial implications of this study are varied. First, this study confirms that using omnichannel retailing can be adopted as an effective response to COVID-19. Given that the COVID-19 pandemic has resulted in enormous shock to the retail and service industry (24), those service providers that currently only operate a single channel may consider introducing omnichannel retailing to provide a better shopping experience and service quality to attract customers (117).

Second, given the high COVID-19 infection and mortality rate, it is important that omnichannel retail service providers (ORSPs) take safety-related strategies that alleviate customers' perceived fears, as well as enhance response efficacy. For example, for the physical channels, ORSPs can place non-contact temperature assessment machines at the entrance to ensure that customers entering the store do not have fever symptoms and arrange for staff to supervise their use of sanitizers. At the same time, ORSPs also need to control the customer flow in time slots to ensure social distancing among customers. However, since such a restriction may lead to long queues, which may conversely exacerbate customers' fears, a possible solution to this problem may be to provide real-time reservation information on their digital channels. Following this, self-efficacy is the next area of improvement for omnichannel retail services. ORSPs can aim to reduce the complexity of the overall system so that consumers can better master the skills required. Also, supportive programs, such as mentorship and demonstrations, should be arranged to engage consumers to improve their confidence in using omnichannel retailing services.

### Limitations and Recommendations

The present study has some limitations, which suggests avenues for future research. The first limitation is the scope of this study. Given that the sample used in this study was collected in Seoul, South Korea, it may prove challenging to directly apply the implications to other cultural or geographical settings. In addition, the majority of the survey respondents were rather young (only 2% were above 50 years old), which may cause the dataset to be questionable in terms of the representativeness of the sampled population. Therefore, further studies that endeavor to overcome the generalizability issues of this study are encouraged. For example, future research may conduct a cross-country study and employ a boosted sample from the older generations to validate our findings. Another limitation is that although this study identified several important factors regarding the determinants of consumers' intentions to use omnichannel retailing, the research model only considered two theoretical lenses: PTM and E-EPPM. Therefore, it is recommended that future studies apply other relevant theories, such as attitude theory (118), self-determination theory (119), trust theory (96), or the unified theory of acceptance, and use the Technology 2 Model (120) to study consumers' intentions to use omnichannel retail services.

## Data Availability Statement

The raw data supporting the conclusions of this article will be made available by the authors, without undue reservation.

## Ethics Statement

Ethical review and approval was not required for the study on human participants in accordance with the local legislation and institutional requirements. Written informed consent for participation was not required for this study in accordance with the national legislation and the institutional requirements.

## Author Contributions

FL, MF, LC, MS, and XW have equally participated in conceptualization, literature review, data analysis, and writing of the paper. All authors contributed to the article and approved the submitted version.

## Funding

This work was supported by the National Social Science Fund of China [No. 20BJY131].

## Conflict of Interest

The authors declare that the research was conducted in the absence of any commercial or financial relationships that could be construed as a potential conflict of interest.

## Publisher's Note

All claims expressed in this article are solely those of the authors and do not necessarily represent those of their affiliated organizations, or those of the publisher, the editors and the reviewers. Any product that may be evaluated in this article, or claim that may be made by its manufacturer, is not guaranteed or endorsed by the publisher.
